# The Cognitive Mechanisms of the SNARC Effect: An Individual Differences Approach

**DOI:** 10.1371/journal.pone.0095756

**Published:** 2014-04-23

**Authors:** Arnaud Viarouge, Edward M. Hubbard, Bruce D. McCandliss

**Affiliations:** 1 Laboratoire Psychologie de la Perception, CNRS/UMR8242, Université Paris Descartes, Paris, France; 2 Educational Neuroscience Lab, Department of Educational Psychology, University of Wisconsin-Madison, Madison, Wisconsin, United States of America; 3 Educational Cognitive Neuroscience Lab, Peabody College, Vanderbilt University, Nashville, Tennessee, United States of America; University G. d'Annunzio, Italy

## Abstract

Access to mental representations of smaller vs. larger number symbols is associated with leftward vs. rightward spatial locations, as represented on a number line. The well-replicated SNARC effect (Spatial-Numerical Association of Response Codes) reveals that simple decisions about small numbers are facilitated when stimuli are presented on the left, and large numbers facilitated when on the right. We present novel evidence that the size of the SNARC effect is relatively stable within individuals over time. This enables us to take an individual differences approach to investigate how the SNARC effect is modulated by spatial and numerical cognition. Are number-space associations linked to spatial operations, such that those who have greater facility in spatial computations show the stronger SNARC effects, or are they linked to number semantics, such that those showing stronger influence of magnitude associations on number symbol decisions show stronger SNARC effects? Our results indicate a significant correlation between the SNARC effect and a 2D mental rotation task, suggesting that spatial operations are at play in the expression of this effect. We also uncover a significant correlation between the SNARC effect and the distance effect, suggesting that the SNARC is also related to access to number semantics. A multiple regression analysis reveals that the relative contributions of spatial cognition and distance effects represent significant, yet distinct, contributions in explaining variation in the size of the SNARC effect from one individual to the next. Overall, these results shed new light on how the spatial-numerical associations of response codes are influenced by both number semantics and spatial operations.

## Introduction

One of the prominent representations of numbers takes the form of a mental number line (MNL). Different behavioral measures are assumed to tap into this representation and to reveal different aspects of it. One of these measures, by showing an association between sides of space and magnitude of numbers, is thought to reflect the spatial orientation of the MNL (from left to right in Western cultures [1], [2], [3]). This is the so-called SNARC (Spatial-Numerical Association of Response Codes) effect: during numerical tasks using a two-alternative, forced choice paradigm, participants respond faster to smaller numbers (relative to the numerical range used in the experiment) with left-sided responses, and to larger numbers with right-sided responses. The SNARC effect was first described by Dehaene and colleagues [1], and has been observed and investigated in multiple studies since (see [4], [5], [6] for reviews). Interestingly, the SNARC effect can be observed in tasks that do not require encoding the magnitude of the numbers presented, such as in a parity judgment. This has led researchers to think of the SNARC effect as an automatic association between numbers and space. However, studies that have examined the SNARC effect have tended to focus more on the cognitive mechanisms that underlie these effects, and less on individual differences in these measures of numerical representations. The mechanisms of the SNARC effect are still debated (e.g., [7]), and a better understanding of how individual differences in the SNARC relate to individual differences in numerical and visuospatial processes may shed light on the underlying mechanisms of the SNARC.

To date, no study has examined whether there are stable individual differences in the SNARC effect. Thus, our first question was whether the SNARC effect can be considered as a robust measure of numerical representations, possibly reflecting the strength of number-space associations. If the SNARC does indeed represent a stable individual difference, then it would be important to know how it relates to other cognitive processes, such as numerical comparison and visuospatial processes that also demonstrate stable individual differences. Therefore, our second question was that of the cognitive constructs of the SNARC effect. By studying the relationship of the SNARC effect to other numerical (distance effect) and visuospatial (assessed by two different mental rotation tasks) measures, we probed the cognitive mechanisms underlying this measure.

The distance effect is used to characterize the precision of the MNL by assessing the degree of overlap between the representations of two different numerosities. Behaviorally, the distance effect corresponds to slower reaction times when comparing two numbers separated by a smaller numerical distance [8]. The more precise the MNL, the less participants are impacted by the numerical distance between the numbers to be compared, resulting in a smaller distance effect. There is recent evidence demonstrating the reliability of the distance effect, especially in tasks using non-symbolic numerical stimuli [9], but also, to a lesser extent, in the context of symbolic comparison tasks [10].

Assuming that the SNARC and distance effect are both behavioral indicators of a unified mental number line, we hypothesized that these two measures should share common variance. This correlation has not yet been directly tested in adults. To date, only one study conducted in fifth and sixth graders has been performed [11], and it yielded contradictory results concerning the correlation between the SNARC and the distance effect. In the first experiment, conducted on 110 fifth graders, the authors found a weak but significant correlation between the SNARC and distance effects, as assessed by parity and comparison tasks, respectively. However, a second experiment using the same tasks in another group of 204 children from fifth and sixth grades found no significant correlation between the SNARC and the distance effect. Given these conflicting results, it remains unclear whether the SNARC effect depends on the numerical distance effect or not.

Another open question about the SNARC effect is the degree to which it depends on visuospatial or verbal processes. The majority of the evidence to date suggests that the SNARC depends on interactions between numerical and spatial circuitry in parietal cortex [5]. Converging evidence from behavioral studies in healthy participants, behavioral studies of patients with neglect, and brain imaging studies in healthy adults all support this proposal. Studies of patients with neglect have shown that these patients show similar deficits on spatial and numerical tasks [12], [13]. Numerical cues have been shown to elicit attention-related ERP components similar to those elicited by purely spatial stimuli like arrows [14], [15]. In addition, a recent fMRI-decoding study demonstrated that spatial mechanisms in posterior parietal cortex are used in basic arithmetic tasks. A classifier trained to infer the direction of eye movements (leftward or rightward) from patterns of parietal brain activity generalized without any additional training to an arithmetic task, classifying subtraction as a leftward eye-movement and addition as a rightward eye-movement and demonstrating that arithmetic processes depend on visuospatial ones [16]. Additionally, children with visuospatial disabilities also show an abnormal SNARC effect [17]. However, it has recently been suggested that the SNARC may not depend on visuospatial processes, but instead depends on verbal coding [18], [19]. Proctor and Cho [19] have proposed that the SNARC effect reflects a polarity correspondence effect, where a benefit in reaction times is observed whenever the polarity of the stimulus and the response are congruent (e.g. “large” and “right” share the same polarity, which leads to faster reaction times when large numbers are responded to on the right).

**Figure 1:**
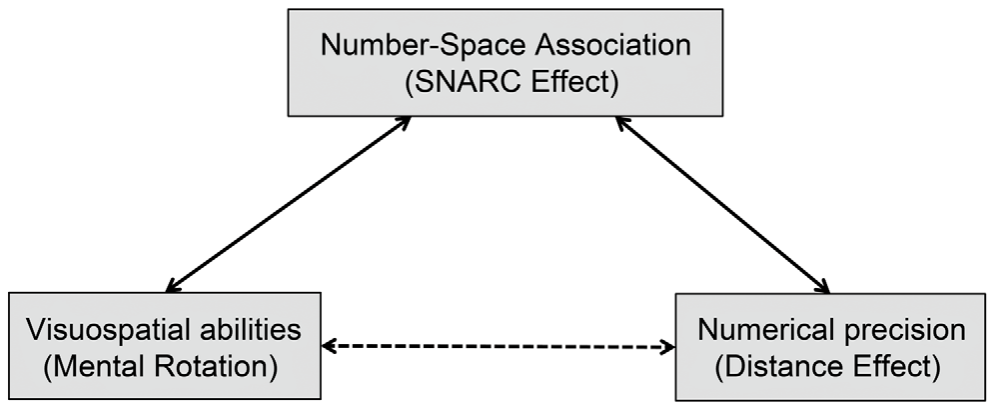
Diagram of the predicted relations. This graph shows the predicted constructs of the SNARC effect according to the account of the spatial mental number line. The connectors show the tested correlations (non-predicted relations are shown with dashed lines).

As one test of the hypothesis that the SNARC effect reflects links between numerical and spatial processes, we predicted that individual differences in the magnitude of the SNARC effect would correlate with visuospatial measures, such as mental rotation, which is thought to rely on parietal circuitry that partially overlaps with parietal mechanisms for numerical processing [5], [20]. The presence of a SNARC effect elicited by irrelevant numerical stimuli in the context of spatial orientation tasks which depend on the dorsal pathway, but not color or shape judgment tasks which depend on the ventral pathway, confirms the neural overlap between numerical and spatial processing and is consistent with our predictions [21], [22]. More specifically, mental rotation performance has been shown to be related to the integrity of white matter tracts underlying the intraparietal sulcus [23], as well as to morphological measures of parietal lobe volume and surface area [24]. Conversely, we predicted no such shared underlying mechanism between the visuospatial tasks and the distance effect, which we assumed to depend solely on numerical codes (see Figure 1 for a graph of the predicted relations).

From a more general point of view, our study seeks to address the question of how number-space associations, as assessed by the SNARC effect, relate to numerical and visuospatial abilities in adult participants. How do those individual differences in what is commonly thought of as a spatialization of numbers relate to the precision of the MNL? And, what is the strength of this relation relative to the relation between the SNARC effect and purely visuospatial tasks? Answering these questions will help us understand the exact nature of the number-space interactions, and their relevance for mathematical abilities in general. This relevance has been shown in the case of purely numerical abilities. For example, stable individual differences in numerical comparison have been shown to correlate with math achievement [25], [26]. However, some studies also suggest a link between visuospatial and mathematical skills. Indeed, links between mental rotation and numerical abilities have been demonstrated in ninth graders [27], and in mathematically gifted adolescent males [28]. It is important to note that while these studies suggest a link between math and spatial abilities in adults, they were mainly conducted in young populations, leaving open the question of how these links change with development. Finally, although the relationship between mental rotation and visuospatial working memory is still debated, a neuroimaging study has indicated a correlation between activity in the parietal lobe and math and visuospatial working memory [29]. Thus, our study will help to shed light on the relevance of the SNARC effect as an individual measure of the strength of number-space associations. Further, by looking at how individual differences in the amplitude of the SNARC effect relates to individual differences in both numerical and visuospatial tasks, we hope to better understand the relationships between visuospatial and mathematical abilities.

## Methods

This project was conducted under the approval of the committee of Vanderbilt University Institutional Review Board (IRB# 090402). All recruited participants gave written informed consent before taking part in this two-session study.

### 1. Participants

Forty-eight participants were recruited through the Vanderbilt University online Psychology research sign-up system. All participants received compensation for their participation. Participants were all right-handed, native English speakers, with normal or corrected-to-normal vision, and were naïve to the study hypotheses. Forty-one of them completed the two experimental sessions, including five participants whose data had to be removed due to mistakes in the order of the experimental blocks (two subjects), partial loss of the data (one subject) or a high (more than 25%) error rate (two subjects). Thus, the sample reported here includes thirty-six participants (age 18 to 31, mean age: 21.6 years, 16 males).

### 2. Tasks

Four tasks were used: a parity task (odd/even judgment on visually presented Arabic digits), a symbolic comparison task (“compare the presented number to a reference number”), and two mental rotation tasks (a 3D block matching task, and a 2D normal vs. mirror letter recognition task). In all four experiments, although stimulus presentation was untimed, participants were instructed to give their response as quickly and as accurately as possible.

#### 2.1. Parity task

Stimuli were Arabic digits between 1 and 9, excluding 5, presented in 24 pt. Microsoft Sans Serif font. On each trial, participants were asked to indicate the parity of the number presented in the middle of the screen, as quickly and as accurately as possible by pressing either the most leftward or the most rightward button of the response box using their left and right index fingers. All visual stimuli were presented in black on a light gray background. For each trial, a fixation point (black dot) appeared in the center of the screen for 500 ms, followed by the target digit, which disappeared as soon as participants responded (no maximum duration was imposed for the response). There was an inter-trial interval (ITI) of 1 s between each experimental trial. The experiment was divided into four blocks between which the participants were allowed to take a short break. The position of the hands on the buttons remained constant throughout experiments and across participants (right index finger on the right button, left index finger on the left button). The assignment between the parity and the response buttons was switched after the first two blocks, and the order of the parity-to-response button assignment was counterbalanced across the participants. To help reinforce the response button mapping, blocks 1 and 3 each started with 8 practice trials (each of the 8 digits presented once in a random order). During the practice trials, the participants were given feedback on their accuracy during the 1 second ITI between trials.

Each of the four blocks began with the presentation of 8 warm-up trials that were identical to the actual trials, consisting of the 8 experimental digits in a random order. These warm-up trials were not included in the analyses, and were used by the experimenter to ensure that the participants were responding as quickly and as accurately as possible. If the experimenter noticed that the participant responded too slowly or inaccurately during these warm-up trials, he/she briefly reminded the participant of the instructions. After these warm-up trials, each of the 8 experimental digits was presented 20 times, resulting in a total of 160 trials per block, randomized within each block.

#### 2.2. Comparison task

The design of the comparison task was identical to the design of the parity task, with four blocks comprising a total of 640 experimental trials. The only difference from the parity task was the instructions. Participants were asked to decide as quickly and accurately as possible whether the presented digit was more or less than 5 by pressing the left or right response button with the corresponding index finger. The order of the assignment between the responses and the buttons was counterbalanced across participants and across the two groups defined by the parity task button-mapping orders (Table 1).

**Table 1:** Experimental design. Each session began with a parity task, followed by a comparison task (Session1), or two mental rotation tasks (Session2).

Order 1	Session 1	Parity-Order 1	Comparison-Order 1	
	Session 2	Parity-Order 1	MR1	MR2
Order 2	Session 1	Parity-Order 1	Comparison-Order 2	
	Session 2	Parity-Order 1	MR2	MR1
Order 3	Session 1	Parity-Order 2	Comparison-Order 1	
	Session 2	Parity-Order 2	MR1	MR2
Order 4	Session 1	Parity-Order 2	Comparison-Order 2	
	Session 2	Parity-Order 2	MR2	MR1

#### 2.3. 3D Mental Rotation task

This task was adapted from the Vanderberg rotation test [30]. The objects used as stimuli were 3D block designs used by Shepard and Metzler [31]. In our task, we selected 30 pairs of 3D block designs from the Vanderberg rotation test. Half of the stimuli consisted of matching pairs (same objects differing by a rotation around the Y axis) the other half consisted of non-matching pairs (one object and its rotated mirror image). One point was given for each correctly answered pair, resulting in a maximum 3D mental rotation score of 30.

Each trial consisted of a 1 sec fixation point, followed by the presentation of the pair of objects, which remained on the screen until the participant indicated their response by pressing one of the two buttons on the response box (the right button was always assigned to the “matching” response). Although no maximum duration was imposed, the participants were instructed to respond as quickly and accurately as possible. A practice trial containing one example of a non-matching pair of objects was presented prior to the 30 experimental trials. Feedback was given and in the case of an incorrect answer, the same trial was presented again, and the participants were invited to look at the two pictures again to understand why the two objects were different. This 3D mental rotation task allowed us to obtain a general score of visuospatial processing for each participant, and to detect possible outliers in the group of participants with respect to visuospatial abilities.

#### 2.4. 2D Mental Rotation task

This 2D mental rotation task was adapted from Cooper and Shepard [32]. Participants were asked to judge whether a presented stimulus was a letter or a mirror image of a letter by pressing one of the two response buttons used in the other tasks described above. The experiment was divided into 4 blocks. Only one letter was used within each block. The letters F, G and R were used for the experimental blocks, the letter L was used during the first block for practice. The letters and their mirror images were rotated at 6 different angles (0, 60, 120, 180, 240 or 300 degrees). Each stimulus was presented twice in a random order within a block, resulting in 72 experimental trials. During the practice block, each stimulus was presented once (12 practice trials). Each trial began with the presentation of a fixation point for 500 ms, followed by the presentation of the stimulus, which lasted until participants responded. The right response button was always assigned to the “letter” response and the left button to the “mirror image” response. The presentation of the stimulus was followed either by a 1s feedback screen showing accuracy during the practice trials, or by a 500 ms blank screen during the experimental trials. Before each block, participants were presented with an instruction screen on which they could see an example of the upright letter and its mirror image.

### 3. Material and Procedure

Participants took part in two one-hour experimental sessions, separated by 14 days (+/−3 days depending on participants' availability). The four different tasks were programmed using Paradigm software (Perception Research Systems) on a Dell 3.33 GHz 32-bit personal computer, equipped with a 21” screen. The Psychology Research Tools (PST) response box was used for response collection.

In order to reduce any possible interference between numerical and visuospatial processes, the numerical comparison task was performed during the first session, while the visuospatial mental rotation tasks were performed during the second session. To assess the SNARC effect with sufficient statistical power and equate for the experimental context in which the participants performed both the comparison and the mental rotation tasks, the parity task was performed once at the beginning of each session (see Table 1 for a description of the experimental design). The testing conditions (room and experimental set up) were kept constant across participants. For each participant, the order of the blocks for the parity task was maintained constant across the two sessions. Thus, in addition to allowing for split-half analysis of the SNARC effect across the two sessions, this design also permitted assessing the test-retest reliability of the SNARC effect between the two sessions.

## Results and Discussion

In the following section we present the analyses of each of the computed measures separately, before describing the analyses of their relationships. For each measure, we controlled for possible gender effects. While gender effects have been extensively reported in the context of spatial tasks, especially in mental rotation tasks [33], there is also some evidence for an effect of gender in numerical tasks [34]. In our data, this effect was absent in the SNARC (t(34)  =  .39, p  =  .7), the numerical distance (t(34)  =  .15, p  =  .88) and the angle effect from the 2D mental rotation task (t(34)  =  .34, p  =  .74) thus justifying our choice to collapse across gender. An effect of gender was found in the 3D mental rotation task. We took this effect into account in our analyses of the relationship between 3D mental rotation and the SNARC effect, as described below.

### 1. Analysis of the SNARC effect

Only correct trials with reaction times between 150 and 1200 ms were included in the analysis, as previously used in SNARC studies [35]. For each participant, more than 85% of the total number of trials passed this criterion. We first calculated, for each participant and each of the 8 numbers tested, a measure of the difference in reaction times (“dRT”), relative to the position of the response-button (dRT  =  mean RT for right responses– mean RT for left responses). The amplitude of the SNARC effect for each participant was then computed as the slope of the linear regression on the dRTs [36], [37]. A negative slope indicates the presence of a SNARC effect (since it shows an advantage for left-sided over right-sided responses for small numbers and an advantage for right-sided responses for large numbers). Using this method, the SNARC effect was assessed for each session separately across the participants. One participant was an outlier regarding the regression slope obtained for the second session, with a regression slope more than 3 standard deviations from the mean, and was removed from further analyses. T-tests were then performed on the obtained regression slopes to test whether the slopes were significantly different from 0. The t-tests performed on the regression slopes showed a significant SNARC effect within each session across the 35 participants (Session 1: t(34)  =  −4.1, p < 0.001, Session 2: t(34)  =  −3.33, p < 0.01). This result replicated previous findings and confirmed the presence of a global SNARC effect in our group of participants, even when they participated in the same experiment during a second session.

### 2. Analysis of the distance effect

The distance effect was obtained by analyzing the data from the comparison task. As with the parity task, only correct trials with reaction times between 150 and 1200 ms were included. In order to calculate the amplitude of the distance effect, we grouped the trials based on the absolute value of the distance to the reference digit 5, and computed the average reaction time for the four distances (1, 2, 3 or 4), in each participant. The amplitude of the distance effect is given by the slope of the regression line when performing a regression analysis on the four average reaction times for each participant. A negative slope shows a decrease in reaction times as the distance between the target and the reference digit increases, which is characteristic of the distance effect. All participants showed a decrease in their average reaction time with increasing numerical distance. A t-test performed on the 35 slopes showed a significant distance effect across our group of participants (mean slope  =  −15.4, t(34)  =  −15.2, p < .001).

### 3. Analysis of the mental rotation tasks

For the 2D mental rotation task (normal vs. mirror letters), a measure of the effect of the angle of rotation on the participants' RTs was computed [32]. To do so, the stimuli were first grouped by the absolute value of their angles of rotation from the corresponding letter shape. Then, for each participant, the average RT for correct responses was computed for each group of stimuli. A regression slope was then calculated, providing a measure of the interaction between the absolute value of the angle of rotation and RTs. One participant showed a regression slope greater than the average plus 3 standard deviations and was not included in further analyses. For the 34 remaining participants, the average regression slope was 3.44 ms/deg, with a standard deviation of 2.12. All participants' regression slopes were significantly greater than 0 (t(33)  =  9.464, p < .001), showing a consistent impact of angle on reaction times. Thus, these slopes were taken as a measure of mental rotation abilities in 2D.

For the 3D mental rotation task, a simple score was computed for each participant, corresponding to the number of correct answers given. This resulted in a total score out of 30. The scores of the 34 participants included in the final analyses ranged between 13 and 27 (mean  =  21.5, std  =  4.05). No univariate outliers were detected using a criterion defined by the mean score ± 3 standard deviations. The average reaction time across all correct answers was also computed for each participant. Across all 34 participants, the average RT was 6800 ms, with a standard deviation of 3501 ms. In order to integrate both accuracy and reaction times in one single measure, and to account for possible speed-accuracy trade-offs, a global Z-score was computed for each participant. This computation was done by averaging the Z-scores based on accuracy and RTs, with the highest Z-scores corresponding to the highest accuracy and fastest RTs (mean  =  0.0, standard deviation  =  0.58). Male participants showed a significantly higher Z-score than female participants (t(32)  =  2.67, p  =  .012). When analyzing reaction times and accuracy separately, we found that this effect was driven by significantly shorter reaction times in the male group (t(32)  =  −2,081, p  =  .045), in the absence of a significant difference in accuracy (t(32)  =  .89, p  =  .38).

### 4. Links between the different tasks

For each of the correlation analyses, we computed Cook's distances based on linear regression to identify possible bivariate influential data points. We then removed, for each analysis separately, the participants showing a Cook's distance greater than the usual convention of 4/N (in our case 4/N  =  0.118, with N  =  34) [38]. We then ran the correlation analyses on the new subset of participants (these analyses were also conducted using the correlation coefficient for the regression analyses of the SNARC instead of the slopes, as suggested in [39], leading to a similar pattern of results).

#### 4.1. Reliability of the SNARC effect

**Figure 2:**
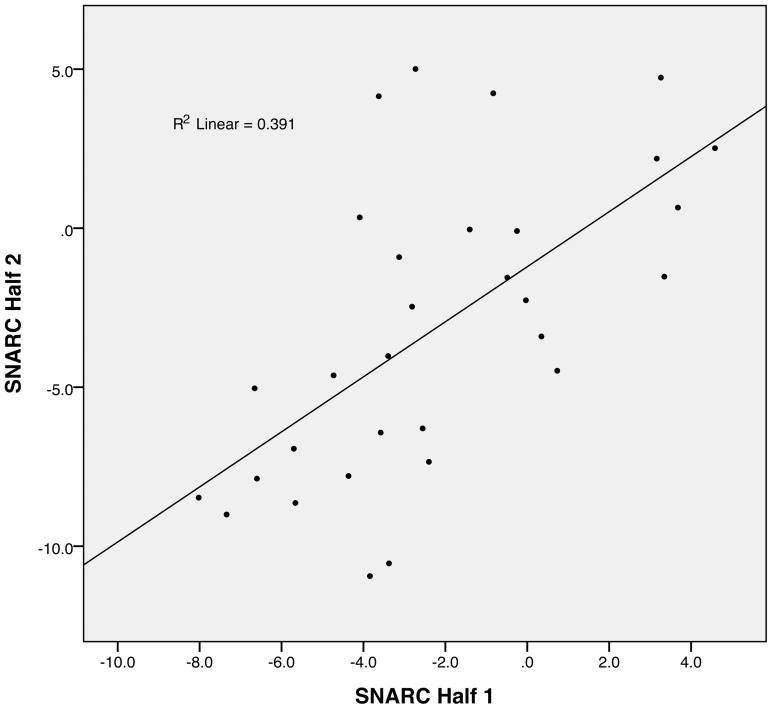
Split-half reliability of the SNARC effect. This graph shows the scatterplot of the correlation between the amplitudes of the SNARC effect computed from two halves of the trials, and the corresponding regression line.

First, we performed a split-half reliability analysis across the two sessions. To do so while preserving the balance in the weights for each number stimulus, we constructed the two “halves” of trials by considering each stimulus separately and alternatively assigning each trial in their order of presentation to each of the two sets of trials. Two SNARC slopes were calculated for each participant based on the two halves of trials defined above (a SNARC effect was also found when considering the two halves of the trials across the two sessions, t(34)  =  −3.83 and −4.24, p < 0.01). A linear regression between the two sets of slopes was performed and the analysis of Cook's distance identified three data points as possibly influential (D  =  .295, .203 and .298 greater than 4/N, with N  =  34). After removing those three participants, a significant correlation was found between the two sets of trials, with a Pearson's r  =  .63 (p < 0.001, two-tailed), showing that the SNARC effect is a robust individual differences measure (Figure 2). The correlation remained significant even when including the three participants identified by Cook's D as possibly influential (r  =  .545, p  =  .001).

**Figure 3:**
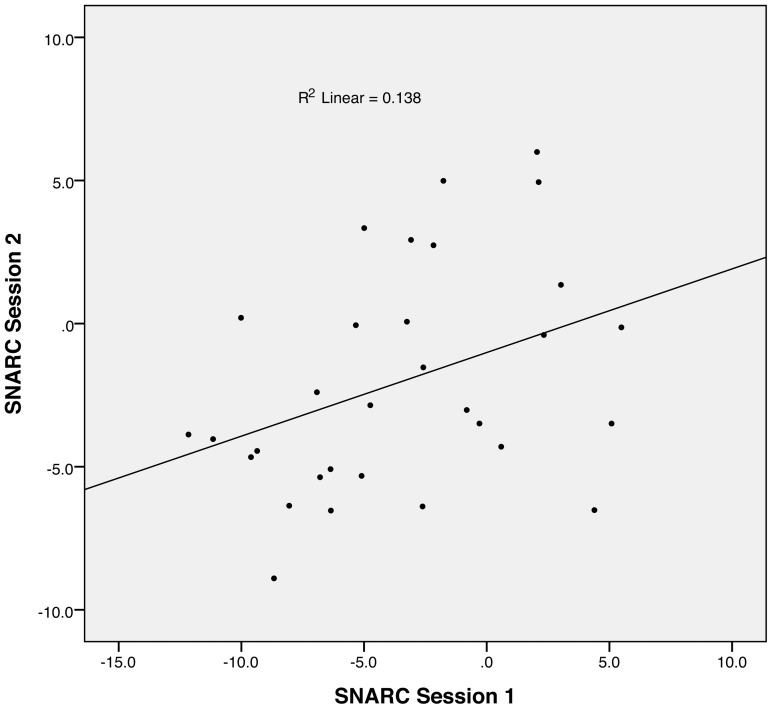
Test-retest reliability of the SNARC effect. This graph shows the scatterplot of the correlation between the amplitudes of the SNARC effect computed from the two experimental sessions, and the corresponding regression line.

We performed the same analysis between the slopes of the SNARC effect obtained for session 1 and session 2. Three participants were first removed based on Cook's distance (D  =  .18, .21 and .25). On the remaining subset of participants (N = 31) we observed a significant correlation between the amplitudes of the SNARC obtained in the two sessions (r  =  .372, p  =  .04; Figure 3). When adding back the three participants identified as possible influential data points, the correlation did not reach significance (r  =  .274, p  =  .116), although showing a trend towards a test-retest reliability of the SNARC effect (1-tailed p-value  =  .058). Given this demonstrated reliability, in the rest of the analyses, we will use the mean amplitude of the SNARC effect between the two experimental sessions.

#### 4.2. Relationship between the SNARC and the distance effect

**Figure 4:**
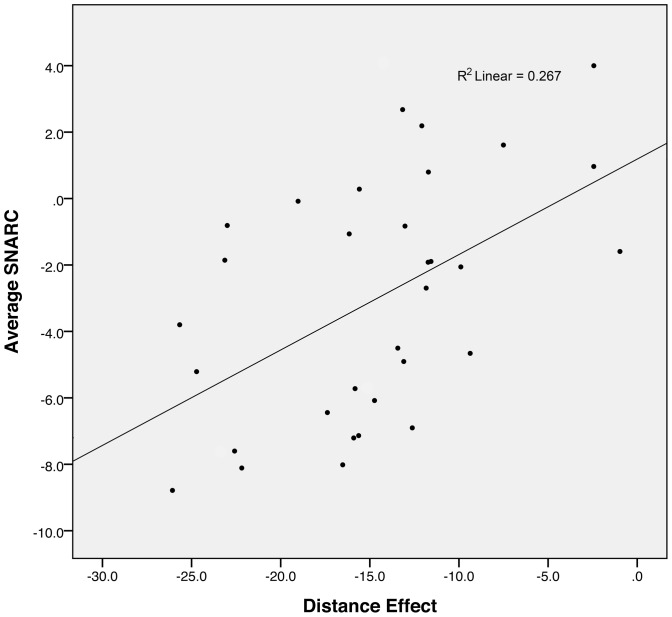
Correlation between the SNARC and the distance effect. This graph shows the scatterplot of the correlation between the mean amplitudes of the SNARC effect and the amplitude of the distance effect, and the corresponding regression line.

When performing a linear regression using the amplitude of the distance effect to explain the variance in the mean amplitude of the SNARC, two participants showed Cook's distances above the 4/N (N = 34) criterion (D  =  .12, and .19). After removing these two participants, we observed a significant correlation between the mean amplitude of the SNARC effect and the amplitude of the distance effect (Pearson's r  =  .516, p  =  .002; Figure 4). The correlation remained significant even when including the two outliers (r  =  .444, p  =  .009). Given the link between the magnitude of interference effects and mean RTs [40], we ran the same correlation controlling for the average RT observed in the comparison task. This partial correlation was also significant (r  =  .466, p  =  .006). This correlation reflects the fact that participants showing a stronger association between numbers and side of response also showed a larger influence of the numerical distance on their reaction times when performing the comparison task.

#### 4.3. Relationship between the two mental rotation tasks

We first ran analyses to test for the correlation between our two mental rotation tasks. The computed average Z-score in the 3D mental rotation task was not significantly correlated with the angle effect extracted from the 2D mental rotation task (r  =  .119, p  =  .504). Although average RTs in the 3D task were significantly correlated with the amplitude of the angle effect in the 2D task (r  =  .413, p  =  .015), this correlation did not hold when controlling for RT in the 2D task, showing that the participants with the fastest reaction times in the 3D block-matching task were also the fastest in the 2D mental rotation task. Overall, our analyses suggest that both tasks tap into distinct variance in the ability to manipulate visuospatial information [41].

#### 4.4. Relationship between SNARC effect and mental rotation tasks

We then tested for a correlation between the average amplitude of the SNARC effect across the two sessions and the two mental rotation measures. A linear regression analysis using the average amplitude of the SNARC effect as the dependent variable allowed extracting Cook's distances for each mental rotation measure separately and thus identifying the corresponding bivariate outliers.

**Figure 5:**
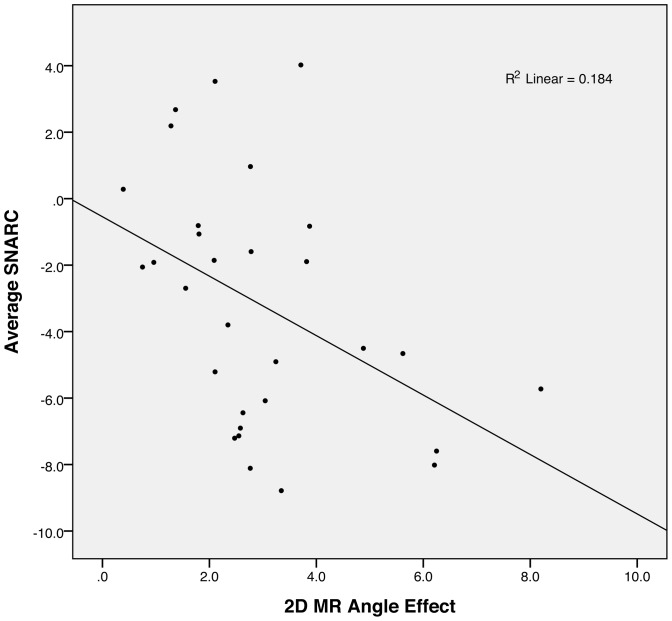
Correlation between the SNARC effect and the 2D Mental Rotation. This graph shows the scatterplot of the correlation between the mean amplitudes of the SNARC effect and the amplitude of the angle effect observed in the 2D mental rotation task, and the corresponding regression line.

Using this method, four participants appeared as possible outliers for the correlation between SNARC effect and 2D mental rotation (D  =  .12, .13, .19 and .23 greater than 4/N, with N  =  34). After removing those four participants from the data, we observed a significant correlation between the mean amplitude of the SNARC effect across the two sessions and the angle effect in the 2D mental rotation task (r  =  −.429, p  =  .018; Figure 5). A trend towards significance was observed when controlling for the average reaction times of the participants in the mental rotation task (r  =  −.321, p  =  .089). When we included the four outliers however, the correlation analysis, although still in the same direction, failed to reach significance (r  =  −.227, p  =  .196). This correlation shows that the participants with greater mental rotation processing speed have a weaker SNARC effect.

Notably, we found no correlation between the mean amplitude of the SNARC effect and any of the three measures extracted from the 3D mental rotation task (r  =  −.160, p  =  .367 for accuracy, r  =  −.279, p  =  .110 for average RT and r  =  .102, p  =  .567 for average Z-score). This relationship was absent in both gender groups separately (r  =  −.23 on 15 males, r  =  .12 on 19 females). Additionally, adding gender as a covariate in a univariate general linear model analysis showed a non-significant interaction between gender and the 3D mental rotation Z-score in the model (F(1,30)  =  0.69, p  =  .41), confirming that gender did not mask the relationship between the SNARC effect and the 3D mental rotation task.

#### 4.5. The cognitive constructs of the SNARC

To compare the relative contribution of the distance effect and the 2D mental rotation task in explaining the variance in the amplitude of the SNARC effect, we performed a multiple regression analysis on the mean amplitude of the SNARC effect, entering the 2D mental rotation slopes and the distance effect in two different models. We first removed five participants identified as potential outliers according to Cook's distance from the linear regression between SNARC effect and either the distance effect or the 2D mental rotation (leading to a final sample of participants N  =  29). This analysis showed that both the distance effect and the 2D mental rotation task were accounting for significant portions of the variance in the amplitude of the SNARC effect. The first model including only the 2D mental rotation measure was significant (R^2^  =  .18, p  =  .022), and the second model including mental rotation and the distance effect elicited a significant change in R^2^ (R^2^  =  .44, ΔR^2^  =  .261, p  =  .002). Across the same group of participants, there was no significant correlation between the distance effect and the 2D mental rotation task (r  =  −.015, p  =  .94). Even when considering the initial data set (34 participants), none of the mental rotation measures (from the 2D and 3D tasks) showed a significant correlation with the distance effect (r  =  .004, p  =  .982 with 2D mental rotation angle effect, and r  =  −.031, p  =  .860 with the average Z-scores from the 3D mental rotation task).

Taken together, these results show that both the distance effect and the 2D mental rotation task contribute to explain separate portions of the variance in the amplitude of the SNARC effect.

## Discussion

In both experimental sessions, our participants showed significant SNARC and distance effects, replicating previous studies. The 2D mental rotation task also replicated a well-known effect of rotation angle on participants' reaction times when asked to judge whether symbolic stimuli were letters or mirror images of the same letters.

Our first question was whether the SNARC effect constituted a robust individual measure. A test-retest analysis between experimental sessions showed only a trend towards significance, with a non-significant decrease in the amplitude of the SNARC effect between the two sessions. It is possible that, even though the sessions were separated by two weeks, the SNARC effect can be reduced with training when participants are tested again under identical experimental conditions. However, our split-half reliability analysis showed a significant correlation, strongly supporting, for the first time, that the SNARC effect reflects a stable individual difference.

When performing further analyses on the average amplitude of the SNARC effect across the two experimental sessions, we found several interesting results.

First, while 3D mental rotation RTs were not significantly correlated with the amplitude of the SNARC effect, we observed that the SNARC was correlated with individual differences in a 2D mental rotation task. Participants who exhibited a greater SNARC effect were also more impacted by the degree of rotation when performing a letter vs. mirror image decision task. If we think of the SNARC effect as interference between numerical and spatial codes, we see here that participants showing greater visuospatial abilities in the 2D mental rotation task are less impacted by the interference between numerical and spatial codes during a parity judgment. These results suggest that poor ability to manipulate mental images is related to poor ability to move attention along the MNL, and therefore a larger SNARC effect.

While the absence of correlation between the 2D and 3D mental rotation tasks is consistent with the fact that 2D, and not 3D mental rotation task showed shared variance with the SNARC effect, several factors may explain this result. 2D and 3D mental rotation tasks tap into three of the five prominent factors of spatial abilities, as revealed by factor analytic methods [41]. The 2D mental rotation task is thought to tap into spatial relations and the 3D mental rotation task into visualization and spatial orientation. It is possible that manipulation of information along the mental number line is more similar to mental rotation in a 2D plane than to manipulation of three-dimensional objects, whereas 3D mental rotation might rely on representations of more complex objects. This idea is supported by a recent study showing distinct neural correlates for 2D and 3D mental rotation [42]. In particular, Kawamachi and colleagues observed that while 3D mental rotation activates regions of the right dorsal premotor cortex, 2D mental rotation of the same objects in a plane activates regions of the parietal cortex, which are thought to be crucial for the representation of ordered numbers on a number line. Future studies may need to examine these issues by systematically varying both 2D and 3D rotation angle.

Second and to a greater extent, the average amplitude of the SNARC effect was significantly correlated with the amplitude of the distance effect. This result supports the idea that both the SNARC and the distance effect are characterizing different aspects of the same representation for numbers, the MNL. Assuming that the amplitude of the SNARC effect reflects the degree to which the participants rely on the spatial representation of the MNL when processing numbers, this correlation shows that the tendency to rely on this spatial representation positively correlates with the impact of numerical distance in comparison tasks. The distance effect is usually thought to decrease with higher precision of the MNL. Our results show that participants with a stronger SNARC effect also have a greater distance effect, that is, a less precise representation of numbers. Again, when considering the SNARC effect as an interference effect, this result would suggest that greater math abilities are associated with a weaker SNARC effect. This interpretation is in agreement with the results of one of the experiments from Dehaene and colleagues [1], who observed a reduced SNARC slope in a group of participants with a scientific background, as compared to a group with a literary background.

Taken together, the correlation between the SNARC and distance effects described above suggests that greater interference due to incongruent response mappings (greater SNARC effect) goes along with a less precise MNL (larger distance effect) and slower mental rotation times.

It could be argued that our SNARC task is similar to the comparison task from which the distance effect was extracted, but also to the 2D mental rotation task in terms of experimental design (central presentation of the stimuli in four blocks with lateralized responses on a keyboard). The measures extracted from these tasks are also similar (regression slopes based on numerical or rotation magnitude). In the papers investigating the reliability of the distance effect, the role of experimental context is highlighted by the authors, suggesting that the reliability of this numerical effect is dependent both on the format (digit vs. non-symbolic quantities, [10]) and on the kind of process engaged in the numerical task (implicit vs. explicit processing, [9]). It is possible that the strength of the correlation observed in our study relates to the similarity of the tasks used to assess the SNARC and the distance effect, as well as the 2D mental rotation angle effect. However, the observed pattern of correlations cannot be solely attributed to these factors, since the distance effect and the 2D mental rotation angle effect were not correlated. Most importantly, multiple regression analyses verified that these two cognitive measures could account for significant and distinct parts of the variance in the SNARC effect, thus constituting two possible cognitive constructs of the SNARC. This result is theoretically important in light of the current debate regarding the role of visuospatial processes in the SNARC effect, as it provides additional support to the contention reviewed above that visuospatial processes play a key role in the SNARC. The correlation between the magnitude of the SNARC effect and mental rotation slopes suggests that the SNARC depends, at least in part, on visuospatial processes. This result is in agreement with a previous study by Herrera and colleagues [43], where they observed a decrease in the SNARC effect when the participants had to maintain visuospatial information while performing a magnitude comparison task, suggesting that visuospatial working memory capacity is important to elicit numerical-spatial interactions such as the SNARC effect. However, unlike in our data, Herrera and colleagues failed to find a correlation between the SNARC and the distance effect. Our results thus weaken the dissociation between the SNARC and the distance effect suggested by Herrera and colleagues.

In two recent studies, Van Dijck and colleagues [44], [45] demonstrated the importance of the experimental context in which the SNARC effect was tested. Using a dual task paradigm, they showed that a verbal working memory load reduced the magnitude of the SNARC in the context of a parity task, while a visuospatial working memory load reduced the magnitude of the SNARC in the context of a magnitude judgment. They therefore suggest that the SNARC observed in parity tasks relies on verbal processes while the SNARC effect observed in magnitude judgments relies on spatial processes. This result is in agreement with the findings of Herrera and colleagues described above, where the “magnitude-SNARC” was shown to be linked to visuospatial working memory. This result was also further confirmed by a principal component analysis, which placed the magnitude-related and the parity-related SNARC effects in two separate components [45].

Given this task-dependence, it is possible that the correlations we observed between the SNARC and visuospatial processes represents a minimal, lower bound on the degree to which individual differences in spatial cognition relate to the strength of number-space associations. If so, we might predict a stronger relation between the “magnitude-SNARC” and visuospatial processes. Unfortunately, we were unable to test this prediction because the SNARC effect calculated from the magnitude comparison task was not significant across our participants. This may be due to the fact that the comparison task was always performed right after the parity judgment, consistent with our observation that the SNARC effect tended to be weaker during the second testing session. One final interesting aspect of these findings is that the distance effect and the spatial measures can be dissociated, which argues against the idea that both effects arise from a single unified representation at the neural level. This idea receives indirect support from a recent ERP study of numerical cuing [14]. They found that spatial effects (as indexed by attention related ERP components) begin only after the peak of the P2p component, which indexes magnitude processing [46].

Taken together, our results shed new light on the construct validity of a spatial-numerical association, the SNARC effect. We demonstrate the robustness of this effect as an individual measure and further demonstrate that variance in the amplitude of the SNARC effect can be explained both by a measure of the precision of the representation of numbers and by a measure of visuospatial abilities. Our results suggest that the SNARC is moderately stable, and is related to individual differences in the degree to which participants use visuospatial coding to represent numerical magnitudes and/or ordered sequences. These results are therefore consistent with the hypothesis that the SNARC effect reflects access to a long-term spatial representation of numbers [5], [20]. To the extent that the SNARC may represent a strategy (as suggested in [47]), it may relate to more general longer-term spatial coding strategies, rather than being purely a result of experimental contexts. Finally, our study makes a larger methodological point, demonstrating the benefit of looking at inter-individual differences to probe questions of cognitive mechanisms.
